# Mutation of the p53 gene precedes aneuploid clonal divergence in colorectal carcinoma.

**DOI:** 10.1038/bjc.1995.46

**Published:** 1995-02

**Authors:** P. J. Carder, K. J. Cripps, R. Morris, S. Collins, S. White, C. C. Bird, A. H. Wyllie

**Affiliations:** Academic Unit of Pathology, University of Leeds, UK.

## Abstract

**Images:**


© 1995 Stockton Press All rights reserved 0007-0920/95 $9.00

### Mutation of the p53 gene precedes aneuploid clonal divergence in colorectal carcinoma

PJ Carder[1], KJ Cripps[2], R Morris[2], S Collins[2], S White[2], CC Bird[2] and AH Wyllie[2]

[1]*Academic Unit of Pathology, Algernon Firth Building, University of Leeds, Leeds LS2 9JT, UK;* [2]*CRC Laboratories, Department of Pathology, University of Edinburgh, Teviot Place, Edinburgh EH8 9AG, UK.*

**Summary** To establish whether p53 mutation precedes or follows clonal divergence in human colorectal carcinomas, 17 tumours were analysed at multiple sites (2-5 each) for single-strand conformation polymorphisms (SSCP) within exons 5-8 of the p53 gene. A previous study had demonstrated subclones of differing DNA ploidy in these tumours, but all showed immunocytochemical evidence for p53 stabilisation, using the monoclonal antibody PAb 1801. Mutations within exons 5-8 of p53 were identified by the presence of an abnormally migrating band in 10 of the 17 carcinomas: five in exon 5, four in exon 7 and one in exon 8. In each of these positive cases, samples from different parts of the carcinoma showed identical gel migration patterns in SSCP analysis. Similarly, the remaining seven tumours were concordant for absence of band shift across all samples of each tumour. Six SSCP-positive cases contained multiple populations differing in DNA ploidy, while four were homogeneously diploid or aneuploid throughout. Very similar proportions were observed in the SSCP-negative cases. In four positive tumours the mutation was confirmed by sequencing or through alteration of nucleotide-specific restriction enzyme cleavage. Identical mutations appeared in every sample from the same tumour. The results provide unequivocal evidence that the same mutant allele of p53 is present throughout each tumour bearing a mutation, regardless of the clonal variation identified by analysis of DNA ploidy. We conclude that in colorectal tumorigenesis mutation of p53 occurs as a single event which precedes and may facilitate the aneuploid clonal divergence of carcinomas.

**Keywords:** colorectal carcinoma; p53; SSCP; clonal evolution

Inactivation of the tumour-suppressor gene p53 is an important step in the development of the majority of human cancers (Hollstein *et al.*, 1991). Functional inactivation occurs most commonly as a result of missense mutation (Baker *et al.*, 1989) but can also result from interaction with oncogenic viral or cellular proteins (Mietz *et al.*, 1992; Momand *et al.*, 1992). The p53 protein is a sequence-specific DNA-binding protein that is active as a transcription factor (Bargonetti *et al.*, 1991) and can interact directly with the replication apparatus (Dutta *et al.*, 1993). There is good evidence that its normal function is to establish $G_1$ checkpoint control in response to DNA damage, so allowing time for DNA repair (Kastan *et al.*, 1991) or the initiation of apoptosis (Clarke *et al.*, 1993). Lack of functional p53 promotes genomic instability (Bischoff *et al.*, 1990; Harvey *et al.*, 1993), which is probably a key factor in acquisition of the multiple 'hits' required for carcinogenesis (Nowell, 1976).

We have recently shown that human colorectal carcinomas containing immunohistochemically detectable p53 are more likely to contain multiple aneuploid DNA stem lines than those which are p53 negative (Carder *et al.*, 1993). This result is in keeping with a relationship between p53 and genomic instability. We also reported that in carcinomas containing stabilised p53 the abnormality was almost always present throughout the tumour even though DNA analysis revealed the presence of divergent stem lines. From these findings we concluded that p53 stabilisation is a critical early event in cancer evolution favouring the development of tetraploid and other aneuploid sublines. Since it is now clear that p53 stabilisation can be the result of the tumour cell environment, independent of mutation (Vojtsek and Lane, 1993), we felt it was important to re-examine these cases for more definitive evidence, firstly, that mutation of p53 had occurred and, secondly, that it preceded clonal divergence in tumour progression. To this end we used the technique of single-stranded conformation polymorphism (SSCP) analysis (Orita *et al.*, 1989; Glavac and Dean, 1993) to identify mutant alleles of p53 and confirm that the same mutant allele was present in all of the samples taken from any one carcinoma, despite independent evidence of clonal divergence between these samples.

Correspondence: AH Wyllie
Received 26 July 1994; revised 18 August 1994; accepted 13 September 1994

### Materials and methods

### *Samples*

Sixty-four samples from 17 colorectal carcinomas from our previously published series (Carder *et al.*, 1993) were studied. An average of four samples per tumour (range 2-5) were analysed and compared with normal colonic mucosa from the same individual. All samples of all tumours contained stabilised p53 as determined by immunohistochemistry using PAb 1801 (Oncogene Science). Flow cytometry was performed on frozen tissue and immunohistochemistry was performed on tissue fixed in periodate lysine paraformaldehyde (PLPD), both as described previously. To minimise possible confusion between tumour and non-neoplastic stroma, samples were assessed as diploid only if tumour cells occupied in excess of 50% of tissue sections from which the corresponding flow cytometric analysis showed a diploid main peak. Samples were considered aneuploid if a separate peak, distinct from the diploid peak, was identified, and aneuploid populations from different samples of the same tumour were considered identical unless they differed in DNA index by more than 0.1. Samples were considered tetraploid if a separate peak with DNA index between 1.9 and 2.1 comprised more than 10% of the nuclei.

### *Single-strand conformation polymorphism (PCR-SSCP) analysis*

The polymerase chain reaction (PCR) was performed on 0.1-1 μg of genomic DNA samples, in an 100 μl reaction containing 200 μM of each deoxynucleotide, 50 pmol of each primer and 2 units of a thermostable *Taq* polymerase in the relevant buffer. PCR was performed in a DNA thermocycler (Hybaid) with the following temperature profile: one cycle of 94°C for 5 min, 30 cycles of 94°C for 1 min, 58°C for 1 min, 72°C for 1 min, and one cycle of 72°C for 10 min. The primers were as follows (5' to 3'): exon 5 up TTCCTCTTC-CTACAGTAGTC and 5 reverse CGATGGTGAGCAGC-TGGG; exon 6 up CCTCACTGATTGCTCTTAGG and 6 reverse CTGAGGTCTGGTTTGCAACT; exon 7 up TGTGTTATCTCCTAGGTTGG and 7 reverse GTCAG-GAGCCACTTGCCA; exon 8 up TCCTATCCTGAGTAG-TGGT and 8 reverse CGAGGTAAGCAAGCAGGA. The samples were extracted once with 24:1 chloroform/isoamylalcohol to remove any mineral oil and 5–10 μl was then denatured in 80 μM sodium hydroxide, 10 μM EDTA, at 48°C for 5 min. Two microlitres sequencing stop solution was added, and the whole sample loaded onto a 5% glycerol, 0.5 × MDE Hydrolink gel (Hoefer Scientific). The gel was run in 1 × TBE on the SE400 PAGE apparatus (Hoefer Scientific) at 25°C, 20 W, for 2–3 h.

Normal and tumour samples from the same individual were run together for comparison. Tumour samples were classified as SSCP positive if a discrete additional band was observed. Bands were visualised by a silver stain (BioRad) as per the manufacturer's instructions with additional washes. The gel was dried on to 3MM paper and laminated.

*Sequence analysis*

A total of nine samples from two cases with band shift on SSCP throughout were sequenced by the dideoxy chain-termination technique as described previously (Cripps *et al.*, 1994) using a commercial kit (Sequenase, Amersham).

*Restriction enzyme digestion using* MspI

A total of eight samples from two cases with band shift on SSCP throughout and known to contain a CGG→CAG mutation in codon 248 were analysed by restriction enzyme digestion using *Msp*I. As described previously (Cripps *et al.*, 1994), disruption of the CCGG recognition site by mutation results in loss of the 135 bp and 168 bp bands and creates an additional 303 bp band.

### Results

Seventeen carcinomas which had been sampled at multiple sites and demonstrated immunmohistochemical positivity for PAb 1801, and for which DNA ploidy values were available, were studied. In 14 cases the majority of nuclei stained intensely. Three cases showed the 'mosaic' pattern, in which positive nuclei were scattered sparsely throughout the tumour. Twelve cases contained multiple clonally distinct subpopulations as determined by assessment of DNA ploidy by flow cytometry, with eight containing two and four containing three variant subpopulations.

From each tumour sample exons 5–8 of the p53 gene were amplified individually and analysed by SSCP. Band shifts indicating the presence of a mutant p53 allele with altered conformation were detected by SSCP in 10 of the 17 carcinomas (59%). An average of four samples per tumour were studied, and the same band shift was present in all samples of each SSCP-positive case, suggesting a single clonal mutation event (Figure 1). The presence of a mutation in the amplified p53 fragment revealing the band shift was confirmed in nine samples of two cases by direct nucleotide sequence analysis. Six samples from one case contained a CGC→CAC mutation in codon 175 and three samples from the other case contained a CAT→TAT mutation in codon 179 (Figure 2). In two further cases a mutation in codon 248 was confirmed in a total of eight samples using the *Msp*I restriction enzyme digestion technique (Figure 3). In all, five mutations occurred in exon 5, four in exon 7 and one in exon 8. Six cases with SSCP-confirmed mutations contained multiple divergent populations as detected by assessment of DNA ploidy, indicating clonal evolution subsequent to mutation (Figure 4). All samples of the SSCP-negative cases were concordant for absence of band shift, confirming that mutation had not occurred (at any rate in this commonly affected part of the p53 gene) during carcinoma progression in these cases. All cases with mutation demonstrated strong positive staining for PAb 1801 in a majority of tumour cell nuclei, but four cases with similarly strong staining were SSCP negative in exons 5–8. The relationship between immunocytochemistry and mutation is described more fully elsewhere (Cripps *et al.*, 1994), in a larger number of cases including the present 17.

**Figure 1** Identical band shifts are revealed by SSCP analysis in all divergent subpopulations of a colorectal carcinoma. N, normal tissue; T1–3, multiple samples from the same carcinoma (DNA index: T1, 1.5/1.6; T2, 1.5; T3, 1.6).

**Figure 2** Dideoxynucleotide sequencing conforms a CAT→TAT mutation in codon 179 (exon 5) in all three samples from this tumour.

**Figure 3** *Msp*I restriction digestion of exons 7–9 confirms a mutation in codon 248 in all tumour samples. Loss of the CCGG recognition site creates an additional 303 bp band and results in loss of the smaller 135 bp and 168 bp bands. The smaller bands are due to contaminating normal tissue.

**Figure 4** Relationship between p53 mutation and clonal evolution. Over all sampled stem lines from any one tumour, band shifts on SSCP analysis of exons 5–8 were either uniformly present (closed symbols) or uniformly absent (open symbols). o, diploid; O, tetraploid; other symbols aneuploid with different aneuploid stem lines within the same tumour being designated by different stem lines. D, diploid; T, tetraploid; $A_1$ and $A_2$, aneuploid.

### Discussion

We have used the technique of single-stranded conformation polymorphism (SSCP) analysis to detect clonality of p53 mutation in colorectal carcinomas. SSCP provided a sensitive method of detecting mutations, as mutant alleles generate characteristic band shifts on electrophoresis under non-denaturing conditions (Orita *et al*., 1989). The technique is sufficiently powerful to allow distinction between sequences differing in a single base pair (Cripps *et al*., 1994). While in theory one might expect a mutant allele to create at least two additional conformers (one from each strand), often only one is observed, and it appears that most new conformers derive from the purine-rich strand (Glavac and Dean, 1993).

Using SSCP we have demonstrated identical band shifts in the same exon in subclones of carcinomas that diverge in DNA ploidy, and also in carcinomas which are homogeneously diploid or aneuploid. We interpret this to indicate that p53 mutation occurs prior to divergence of clones differing in DNA ploidy, and hence any one tumour possesses a single p53 mutation throughout. In a large series of carcinomas reported separately (Cripps *et al*., 1994) we have confirmed by sequencing that band shifts detected by SSCP invariably denote mutation, and that the technique used here identifies more than 70% of all naturally occurring mutations in the region of p53 studied (exons 5–8). It may be argued that identical band shifts might still represent different point mutations within the same amplified fragment. We feel that this interpretation is unlikely for three reasons. Firstly, even minor differences in nucleotide substitution (e.g. in adjacent positions of the same codon) can lead to profound differences in band shift by SSCP analysis using the methods employed here (Cripps *et al*., 1994). Secondly, in the now considerable literature on p53 mutations in cancer, the occurrence of two distinct mutations in the same exon in any one tumour is uncommon. Finally, we have used direct sequencing to confirm identity of mutation throughout in two cases, and in a further two we have used a rapid restriction enzyme digestion technique to confirm identity of mutation site.

The observation that an identical mutation in p53 occurs throughout affected carcinomas, including those with and without clonal divergence in DNA ploidy, provides strong evidence for a single mutational event early in the development of these cancers. Around 20–30% of all colorectal carcinomas containing immunohistochemically stable p53 appear not to contain p53 mutations (Wynford-Thomas, 1993; Baas *et al*., 1994; Cripps *et al*., 1994) but examples of these in this series also showed divergent subclones concordant in p53 immunocytochemistry. Hence, even the non-mutational abnormalities that affect p53 stability may also be an early event. The data presented here emphasise the central role of wild-type p53 in preventing one type of genomic instability. This instability appears to result from continued DNA replication in the presence of DNA damage (Lane, 1992; Livingstone *et al*., 1992; Yin *et al*., 1993) and tends to produce near-tetraploid subclones (Carder *et al*., 1993). It is interesting that the selective growth advantage afforded by this instability appears to be a feature favouring carcinoma rather than adenoma growth, since p53 mutations are unusual in adenomas. Other lesions involved in colorectal tumorigenesis but unrelated to p53, such as mutation in the DNA repair genes implicated in hereditary non-polyposis colon cancer (Peltomäki *et al*., 1993), also initiate genomic instability, albeit of a different type, and associate preferentially with carcinoma rather than adenoma.

In conclusion we establish here by DNA analysis the impression gained from our previous immunohistochemical study: in colorectal tumorigenesis p53 mutation is a critical early lesion occurring as a single clonal mutational event which precedes and probably facilitates the emergence of divergent aneuploid tumour subpopulations.

**Acknowledgements**
We would like to thank Juliet Hamblin for expert typing of the manuscript, Steven Toms for photography and Andrew Hay for the illustrations.

### References

BAAS ID, MULDER J-WR, OFFERHAUS GJA, VOGELSTEIN B AND HAMILTON SR. (1994). An evaluation of six antibodies for immunohistochemistry of mutant p53 gene product in archival colorectal neoplasms. *J. Pathol.*, **172**, 5–12.

BAKER SJ, FEARON ER, NIGRO JM, HAMILTON SR, PREISINGER AC, JESSUP JM, VAN TUINEN P, LEDBETTER DH, BARKER DF, NAKAMURA Y, WHITE R AND VOGELSTEIN B. (1989). Chromosome 17 deletions and p53 gene mutations in colorectal carcinomas. *Science*, **244**, 217–221.

BARGONETTI J, FRIEDMAN PN, KERN SE, VOGELSTEIN B AND PRIVES C. (1991). Wild type but not mutant p53 immunopurified proteins bind to sequences adjacent to the SV40 origin of replication. *Cell*, **65**, 1083–1091.

BISCHOFF FZ, YIM SO, PATHAK S, GRANT G, SICILANO MJ, GIOVANELLA BC, STRONG LC AND TAINSKY MA. (1990). Spontaneous abnormalities in normal fibroblasts from patients with Li-Fraumeni cancer syndrome: aneuploidy and immortalisation. *Cancer Res.*, **50**, 7979–7984.

CARDER PJ, WYLLIE AH, PURDIE CA, MORRIS RG, WHITE S, PIRIS J AND BIRD CC. (1993). Stabilised p53 facilitates aneuploid clonal divergence in colorectal carcinoma. *Oncogene*, **8**, 1397-1401.

CLARKE AR, PURDIE CA, HARRISON DJ, MORRIS RG, BIRD CC, HOOPER ML AND WYLLIE AH. (1993). Thymocyte apoptosis induced by p53-dependent and independent pathways. *Nature*, **362**, 786-787.

CRIPPS KJ, PURDIE CA, CARDER PJ, WHITE S, KOMINE K, BIRD CC AND WYLLIE AH. (1994). A study of stabilisation of p53 protein versus point mutation in colorectal carcinoma. *Oncogene*, **9**, 2739-2743.

DUTTA A, RUPPERT JM, ASTER JC AND WINCHESTER E. (1993). Inhibition of DNA replication factor RPA by p53. *Nature*, **365**, 79-82.

GLAVAC D AND DEAN M. (1993). Optimization of the single-strand conformation (SSCP) technique for detection of point mutations. *Hum. Mutat.*, **2**, 404-414.

HARVEY M, SANDS AT, WEISS RS, HEGI ME, WISEMAN RW, PANTAZIS P, GIOVELLA BC, TAINSKY MA, BRADLEY A AND DONEHOWER LA. (1993). *In vitro* growth characteristics of embryo fibroblasts isolated from p53 deficient mice. *Oncogene*, **8**, 2457-2467.

HOLLSTEIN M, SIDRANSKY D, VOGELSTEIN B AND HARRIS C. (1991). p53 mutations in human cancer. *Science*, **253**, 49-53.

KASTAN MB, ONYKWERE O, SIDRANSKY D, VOGELSTEIN B AND CRAIG RW. (1991). Participation of p53 protein in the cellular response to DNA damage. *Cancer Res.*, **51**, 6304-6311.

LANE DP. (1992). p53: guardian of the genome. *Nature*, **358**, 15-16.

LIVINGSTONE LR, WHITE A, SPROUSE J, LIVANOS E, JACKS T AND TLSTY TD. (1992). Altered cell cycle arrest and gene amplification potential accompany loss of wild type p53. *Cell*, **70**, 923-935.

MIETZ JA, UNGER T, HUIBREGTSE JM AND HOWLEY PM. (1992). The transcriptional transactivation function of wild type p53 is inhibited by SV40 large T-antigen and by HPV-16 E6 oncoprotein. *EMBO J.*, **11**, 5013-5020.

MOMAND J, ZAIBETTI GP, OLSON DC, GEORGE D AND LEVINE AJ. (1992). The mdm-2 oncogene product forms a complex with the p53 protein and inhibits p53-mediated transactivation. *Cell*, **69**, 1237-1245.

NOWELL PC. (1976). The clonal evolution of tumour cell populations. *Science*, **194**, 23-28.

ORITA M, IWAHANA H, KANAZAWA H, HAYASHI K AND SEKIYA T. (1989). Detection of polymorphisms of human DNA by gel electrophoresis as single strand conformation polymorphisms. *Proc. Natl Acad. Sci. USA*, **86**, 2766-2770.

PELTOMÄKI P, AALTONEN LA, SISTONEN P, PYLKKÄNEN L, MECKLIN J-P, JÄRVINEN H, GREEN JS, JASS JR, WEBER JL, LEACH FS, PETERSEN GM, HAMILTON SR, DE LA CHAPELLE A AND VOGELSTEIN B. (1993). Genetic mapping of a locus predisposing to human colorectal cancer. *Science*, **260**, 810-812.

VOJTSEK B AND LANE DP. (1993). Regulation of p53 protein expression in human breast cancer lines. *J. Cell Sci.*, **105**, 607-612.

WYNFORD-THOMAS D. (1993). p53 in tumour pathology: can we trust immunocytochemistry. *J. Pathol.*, **166**, 329-330.

YIN Y, TAINSKY MA, BISCHOFF FZ, STRONG LC AND WAHL GM. (1993). Wild type p53 restores cell cycle control and inhibits gene amplification in cells with mutant p53 alleles. *Cell*, **70**, 937-948.


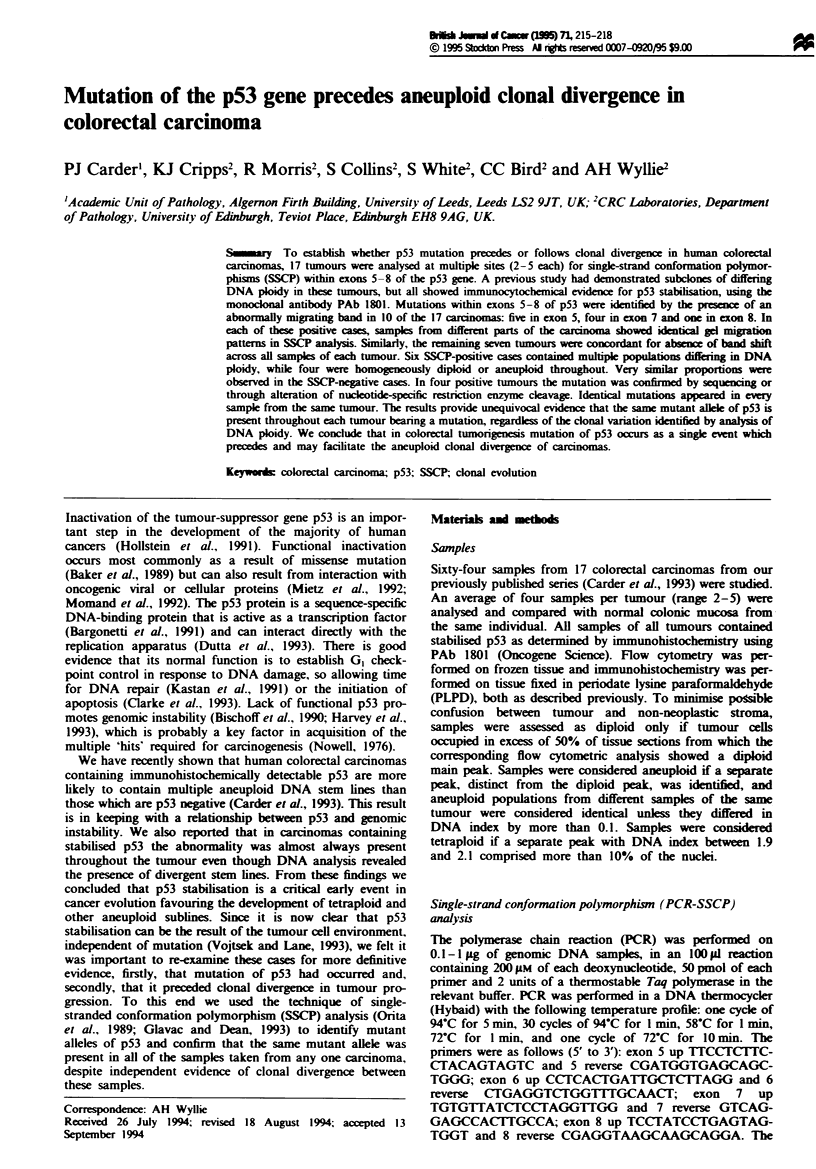

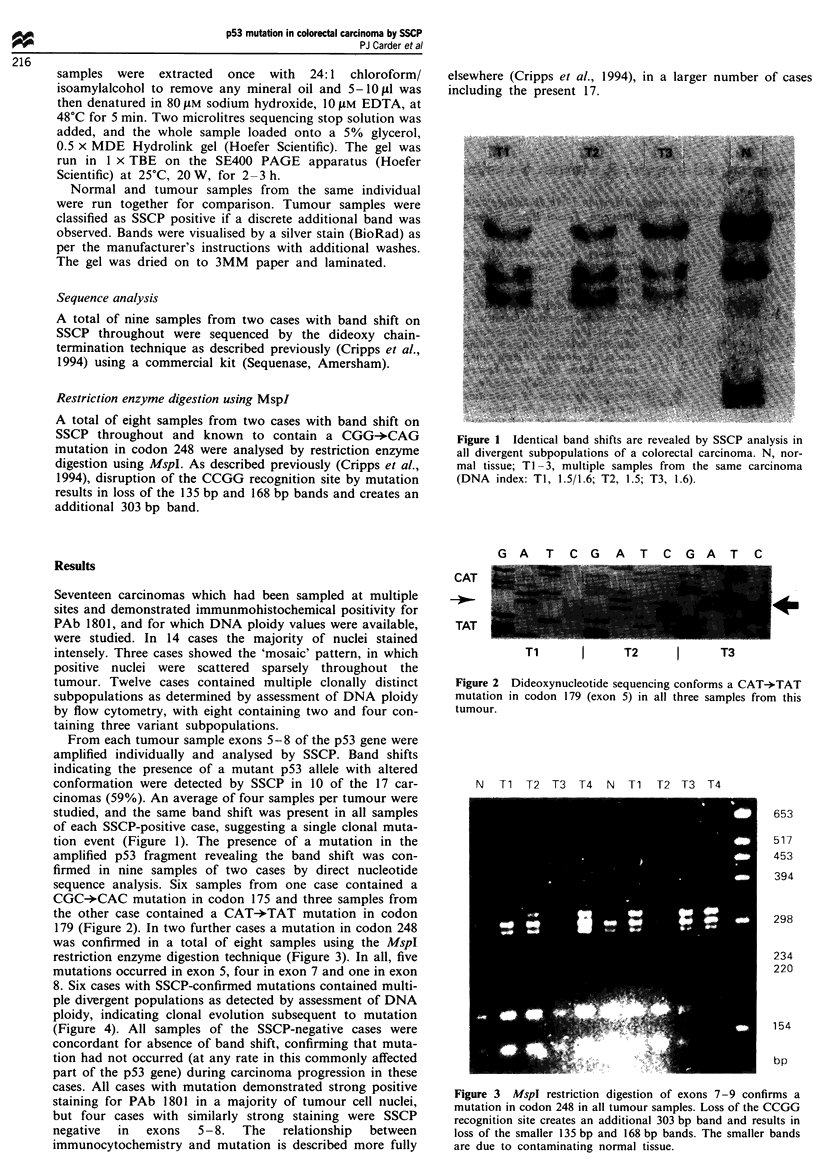

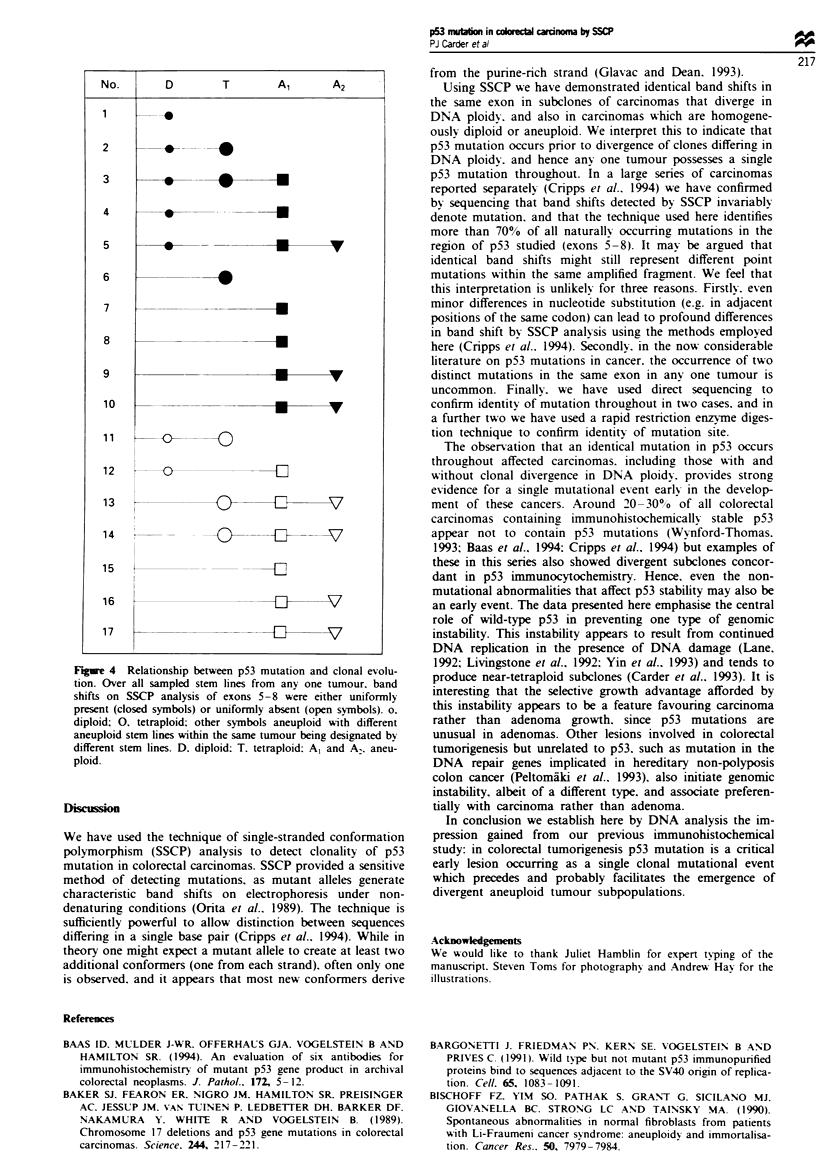

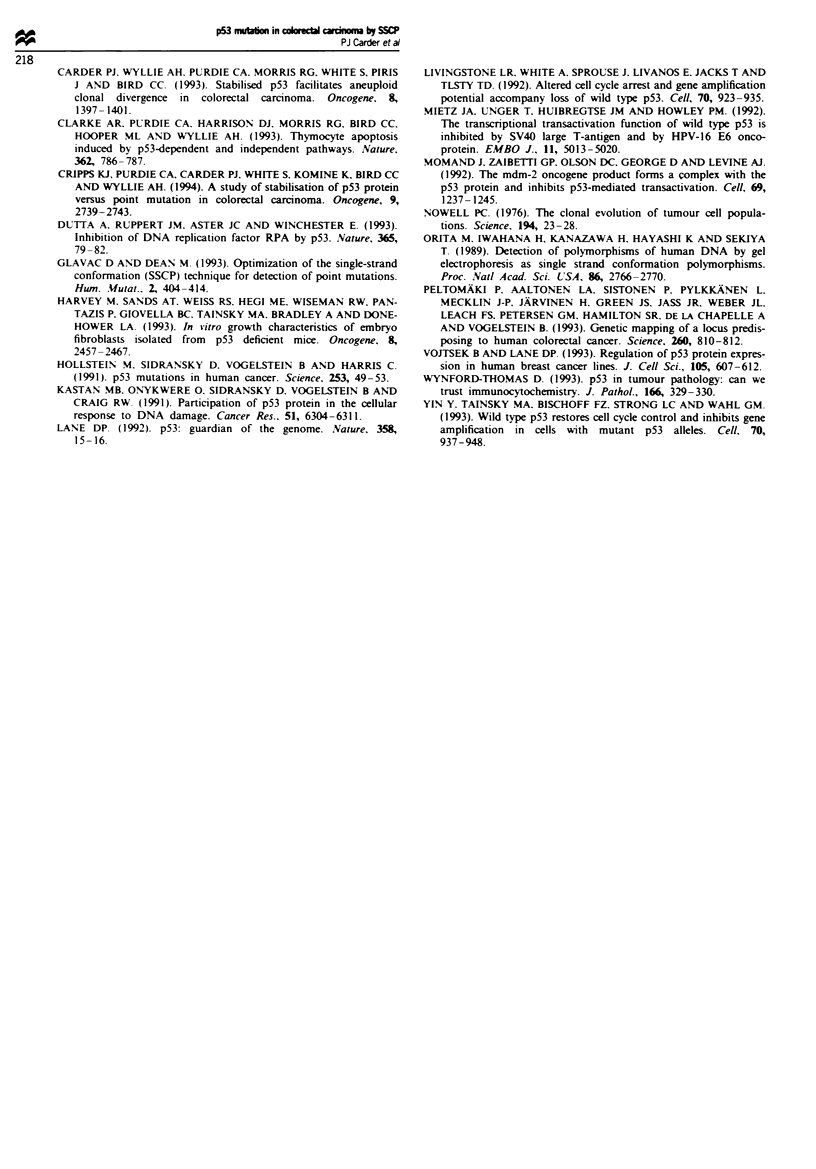


## References

[OCR_00374] Baas I. O., Mulder J. W., Offerhaus G. J., Vogelstein B., Hamilton S. R. (1994). An evaluation of six antibodies for immunohistochemistry of mutant p53 gene product in archival colorectal neoplasms.. J Pathol.

[OCR_00380] Baker S. J., Fearon E. R., Nigro J. M., Hamilton S. R., Preisinger A. C., Jessup J. M., vanTuinen P., Ledbetter D. H., Barker D. F., Nakamura Y. (1989). Chromosome 17 deletions and p53 gene mutations in colorectal carcinomas.. Science.

[OCR_00389] Bargonetti J., Friedman P. N., Kern S. E., Vogelstein B., Prives C. (1991). Wild-type but not mutant p53 immunopurified proteins bind to sequences adjacent to the SV40 origin of replication.. Cell.

[OCR_00395] Bischoff F. Z., Yim S. O., Pathak S., Grant G., Siciliano M. J., Giovanella B. C., Strong L. C., Tainsky M. A. (1990). Spontaneous abnormalities in normal fibroblasts from patients with Li-Fraumeni cancer syndrome: aneuploidy and immortalization.. Cancer Res.

[OCR_00407] Carder P., Wyllie A. H., Purdie C. A., Morris R. G., White S., Piris J., Bird C. C. (1993). Stabilised p53 facilitates aneuploid clonal divergence in colorectal cancer.. Oncogene.

[OCR_00417] Cripps K. J., Purdie C. A., Carder P. J., White S., Komine K., Bird C. C., Wyllie A. H. (1994). A study of stabilisation of p53 protein versus point mutation in colorectal carcinoma.. Oncogene.

[OCR_00425] Dutta A., Ruppert J. M., Aster J. C., Winchester E. (1993). Inhibition of DNA replication factor RPA by p53.. Nature.

[OCR_00428] Glavac D., Dean M. (1993). Optimization of the single-strand conformation polymorphism (SSCP) technique for detection of point mutations.. Hum Mutat.

[OCR_00433] Harvey M., Sands A. T., Weiss R. S., Hegi M. E., Wiseman R. W., Pantazis P., Giovanella B. C., Tainsky M. A., Bradley A., Donehower L. A. (1993). In vitro growth characteristics of embryo fibroblasts isolated from p53-deficient mice.. Oncogene.

[OCR_00442] Hollstein M., Sidransky D., Vogelstein B., Harris C. C. (1991). p53 mutations in human cancers.. Science.

[OCR_00447] Kastan M. B., Onyekwere O., Sidransky D., Vogelstein B., Craig R. W. (1991). Participation of p53 protein in the cellular response to DNA damage.. Cancer Res.

[OCR_00449] Lane D. P. (1992). Cancer. p53, guardian of the genome.. Nature.

[OCR_00453] Livingstone L. R., White A., Sprouse J., Livanos E., Jacks T., Tlsty T. D. (1992). Altered cell cycle arrest and gene amplification potential accompany loss of wild-type p53.. Cell.

[OCR_00457] Mietz J. A., Unger T., Huibregtse J. M., Howley P. M. (1992). The transcriptional transactivation function of wild-type p53 is inhibited by SV40 large T-antigen and by HPV-16 E6 oncoprotein.. EMBO J.

[OCR_00463] Momand J., Zambetti G. P., Olson D. C., George D., Levine A. J. (1992). The mdm-2 oncogene product forms a complex with the p53 protein and inhibits p53-mediated transactivation.. Cell.

[OCR_00469] Nowell P. C. (1976). The clonal evolution of tumor cell populations.. Science.

[OCR_00473] Orita M., Iwahana H., Kanazawa H., Hayashi K., Sekiya T. (1989). Detection of polymorphisms of human DNA by gel electrophoresis as single-strand conformation polymorphisms.. Proc Natl Acad Sci U S A.

[OCR_00483] Peltomäki P., Aaltonen L. A., Sistonen P., Pylkkänen L., Mecklin J. P., Järvinen H., Green J. S., Jass J. R., Weber J. L., Leach F. S. (1993). Genetic mapping of a locus predisposing to human colorectal cancer.. Science.

[OCR_00486] Vojtesek B., Lane D. P. (1993). Regulation of p53 protein expression in human breast cancer cell lines.. J Cell Sci.

[OCR_00490] Wynford-Thomas D. (1992). P53 in tumour pathology: can we trust immunocytochemistry?. J Pathol.

[OCR_00494] Yin Y., Tainsky M. A., Bischoff F. Z., Strong L. C., Wahl G. M. (1992). Wild-type p53 restores cell cycle control and inhibits gene amplification in cells with mutant p53 alleles.. Cell.

